# Normative developmental vs. reverse developmental trends in memory distortion: a framework to investigate the impact of internal and external influences on memory and their relevance to legal decisions

**DOI:** 10.3389/fpsyg.2023.1232753

**Published:** 2023-08-16

**Authors:** Brittany J. Rosendaul, I-An Su, Stephen J. Ceci

**Affiliations:** Child Witness and Cognition Lab, Department of Psychology, Cornell University, Ithaca, NY, United States

**Keywords:** memory distortion, developmental reversals, endogenous sources, exogenous sources, false memories, suggestibility, psychology and law

## Abstract

There are two opposing positions regarding the development of memory: the normative developmental position, and the reverse developmental position. The normative position, which has long been the default presupposition, supports the notion that susceptibility to memory distortion, including false memories, decreases with age. In contrast, the concept of “developmental reversals” supports the notion that susceptibility to memory distortion and false memories increases with age. Each perspective finds support from existing theories as well as from research on endogenous and exogenous sources of influence. In a legal context, having an accurate understanding of the developmental course of false memory can contribute on the one hand to mitigating wrongful convictions and, on the other hand, to appreciating the accuracy of children’s statements when warranted. This review aims to integrate the existing literature regarding these seemingly opposite developmental courses and construct a framework outlining the conditions under which we may observe one age trend over the other. This entails an examination of the paradigms that have been invoked to support these competing positions, specifically developmental responses to internal vs. external sources of distortion.

## 1. Introduction

When it comes to memory, we may intuitively think of a close correlation between accuracy and chronological age, at least until old age when memory declines (Mueller-Johnson and Ceci, 2004; Mueller-Johnson et al., 2007). Children have long been publicly regarded as an unreliable memory source (Wright et al., 2010), made salient by the historical skepticism surrounding child eyewitness testimony dating back to the beginnings of scientific psychology (e.g., Ceci and Bruck, 1993, 1995; Dale et al., 1978; Melton, 1981). Upon asking open-access artificial intelligence to provide “descriptors of children,” two of the ten responses refer directly to children’s “active imaginations” (ChatGPT, 2023), suggesting that the skepticism of former times is still with us.

This line of thinking is congruent with the *normative* developmental view of memory; a developmental framework which suggests that as chronological age increases, we anticipate a *decrease* in susceptibility to memory distortion. This normative developmental position has withstood the tests of time and prevailed as the popular consensus among memory developmentalists since the 1970s (see Ceci and Bruck, 1993 for historical review). It is based on an array of paradigms and independent variables (such as recognition, free recall, cued recall, short-term memory savings, release from proactive inhibition, and so on), and it is often associated with age-related changes in specific cognitive, social, and neurobiological factors that drive performance.

Some of the most prominent support for the normative developmental position comes from neurobiological changes with chronological age. Over the course of childhood, neural functioning develops and strengthens, particularly in regions such as the prefrontal cortex that moderate executive functions such as tracking, monitoring, and inhibition. With these neurobiological developments come improvements in working memory and consequently increased overall cognitive functioning (Constantinidis and Klingberg, 2016).

Predating the exponential growth in neurobiology in recent decades, there has been a steady stream of findings documenting cognitive and social influences on memory development. Moreover, endogenous and exogenous factors, including but not limited to strategy development, emotional valence, and suggestive techniques, were shown to be causally related to memory accuracy and resistance to false suggestions. With rare exceptions, this literature demonstrated normative trends of overall memory improvement with chronological age.

Ceci and Bruck (1993) reported that in 15 out of 18 published experiments, there were both age-related decreases in suggestibility and increases in accuracy, with preschoolers showing the poorest overall performance compared to older children, adolescents, and adults. This normative pattern of increasing accuracy with age has been replicated numerous times over the past several decades, both in individual studies and in syntheses of the literature (e.g., Saywitz, 1990; Zaragoza et al., 1992; Ceci et al., 1995; Ceci and Bruck, 1995; Bruck and Ceci, 1999; Gordon et al., 2001; Goodman et al., 2017; Gonzalves et al., 2022). Across four experiments, Ceci et al. (1987) examined mechanisms driving children’s heightened vulnerability to external sources of suggestion: misleading post-event information; social forces, such as pressures to conform and receiving praise for wrong responses; and provision of false information by an adult authority figure, each of which were found to yield higher rates of memory error in 3- and 4-year-olds than older children, particularly compared to the old*est* children (12-year-olds).

In two of Ceci et al.’s (1987) experiments, social forces were controlled to parse the true causes of children’s suggestibility, and researchers found that accessing the original memory trace was being directly altered by false suggestions. In other words, the experimental design minimized the operation of social forces so that any remaining age-related changes were due to cognitive changes (alteration of the underlying memory trace) rather than desire to please authority figures. This implicates the active role of memory-based mechanisms such as trace attenuation, trace alteration, and retrieval interference. The findings from these studies and many others like them provided early evidence that young children’s memories for events—not just their reports of events, but their underlying memories as well—can be distorted as a result of exposure to post-event information. Such studies were taken as evidence of actual memory impairment as opposed to mere report errors. They formed the basis of the normative developmental position.

Many of the factors that have been investigated in the existing literature have been invoked in support of normative developmental age trends. However, particularly in the face of new research, there is evidence that: (1) these factors and paradigms can produce reverse developmental trends, and (2) there are specific factors that may uniquely predispose older populations to memory distortions. The idea that memory becomes *increasingly susceptible* to distortion in tandem with chronological age forms the foundation of *developmental reversals* (Brainerd and Reyna, 1998).

Much of the evidence supporting developmental reversals is more recent than the evidence supporting normative developmental trends (e.g., Brainerd et al., 2008a,b). The specific emphasis on developmental reversals can be traced largely to the contemporary research produced by Brainerd and Reyna’s (2001, 2002, 2012) analysis of fuzzy-trace theory. Fuzzy-trace theory, or FTT, suggests that memories are not stored verbatim, but rather through conceptual representations, or “gist traces” (Reyna and Kiernan, 1995). With age, comes the ability to rely on such higher-level cognitive scaffolding. Specifically in terms of memory, this gist retrieval has been shown to coincide with increases in false memories (Brainerd and Reyna, 2012).

When children do not yet possess the skillset or semantic knowledge to create these gist traces to aid the encoding, storage, and retrieval of memories, they must rely more heavily on verbatim memory and are therefore less likely to make gist-based memory errors. For example, the question “Did you drink a Coke at lunch?” may cue verbatim or gist traces, or both. Under FTT, we anticipate that the verbatim memory of having had a Coke at lunch will aid in suppressing false memories (e.g., thinking that you had a Sprite at lunch), whereas gist trace aids in matching semantic features (e.g., remembering that you had a cold, fizzy drink at lunch) which may support false memories of other types of cold fizzy drinks. Both verbatim and gist traces contain potentially accurate information (in the sense that you encoded the experienced event) and therefore do not explicitly contribute to memory distortion. When these conceptual representations are over-relied on, we see the outcome of false memories (Brainerd and Reyna, 2012).

In this paper we begin by synthesizing the developmental findings, going back a half century to the classic work of Flavell and his students and show how these findings continue to be relevant in explaining age trends in memory accuracy. In doing this, we organize the literature into developmental acquisitions that are cognitive and social so to identify pre-existing, non-situationally-dependent internal and societal factors that impact all peoples to some degree. We also briefly address neurobiological developments that moderate these acquisitions such as changes in the prefrontal cortex that subserve monitoring, inhibition, and so on. Following this, we organize this research into endogenous and exogenous forms of suggestibility, with the former (endogenous) referring to internally generated sources, such as when in the absence of any external suggestions a lure spontaneously activates semantically related words that were never encountered during the event or when a witness’s internal reveries result in the creation of memory traces as a direct consequence of the revery as opposed to a direct observation. In contrast, exogenous sources of suggestion refer to factors that are encountered via external influences, such as when an interviewer makes a false suggestion or employs leading questions. We ultimately outline a framework with the intention of explaining and anticipating circumstances under which normative vs. reverse developmental trends may emerge.

## 2. Cognitive and social considerations

### 2.1. Knowledge

Knowledge is one of the most significant factors in memory, as it can impact every facet of recollection, including information encoded incorrectly prior to retrieval (e.g., McCutchen, 2000; Dewitt et al., 2012) as well as the retrieval process itself. Within the context of developmental trends, knowledge can cause a chain reaction, so to speak, in which its presence vs. absence can determine the potential for other endogenous and exogenous suggestibility factors. For example, without a knowledge or awareness of social expectations as they pertain to stereotypes, schema, and scripts, these sources of suggestion cannot influence memory.

Although this *may* support normative developmental trends (e.g., when age implicates a greater knowledge of social expectations, which in turn accurately align with an experience and therefore effectively aid in memory storage or retrieval), as the above suggests, neither the possession of knowledge nor its implications are *invariably* age normative. In some situations, a young child may possess greater knowledge than an older child or adult, such as for the characters of their favorite show or the layout of their school. Aspects of familiarity and increased knowledge such as these further supplements a demonstration of developmental reversals. Simultaneously, research has demonstrated that the *lack* of knowledge also affects the capacity to support developmental reversals.

For example, Ceci et al. (2007) implemented the use of multidimensional scaling (MDS) and studied 4- and 9-year-olds’ susceptibility to suggestion in the context of a story about a class trip to a zoo where they observed various animals. When there was a dimension available to categorize the animals that only the older children possessed (e.g., predators, arachnids, avians, etc.), they were *more likely* to misreport that they had seen non-observed animals characterized by these dimensions (e.g., other predators or arachnids they had not actually seen) than were younger children who did not possess these dimensions in the first place, hence never encoded them and never employed them during retrieval.

Such findings suggest that knowledge is capable of both supporting accurate recall as well as impeding it, and as we will demonstrate, these opposing outcomes are each compatible with both the normative and reverse developmental trends; developmental outcomes in both directions are related to children’s knowledge representations and the direction can be predicted by an understanding of their representations. The organization and interconnectedness of the knowledge structure can have a substantial impact on developmental outcomes. When younger children’s representation of knowledge is more elaborate than older children’s, they tend to be more likely to make associative errors, whereas the reverse is true when older children’s knowledge is more elaborate—which is far more common.

### 2.2. Scripts

Another focus of memory researchers has been on the understanding and application of *scripts*. Scripts are temporally organized general knowledge structures that depict the sequence of normally occurring events in their proper temporal order (Fivush et al., 1992). These scripts allow us to form expectations regarding the world around us, which leads to inferential reasoning and gap-filling. The ability to accurately organize and depend on scripts develops in tandem with chronological age, but this does not mean that it ineluctably supports the normative developmental position as we will show.

In some cases, we do in fact see demonstrations of normative development. For example, in cases where a vignette follows the script accurately and the latter is used efficiently to support memory, we see a close positive correlation between chronological age and memory accuracy. As an illustration, Pillemer (1992) exposed 3.5- and 4.5-year-olds attending a preschool program to a fire drill evacuation due to a teacher who burned popcorn. The presence of a script for fire drills varied as a function of children’s age. The older children had all experienced a fire drill in the past, but the younger children had not. Teachers and children exited the building and waited outside while local police and firefighters turned the alarm off and cleared the building for reentry.

Seven years later, these children were interviewed again and asked about the details of that day. The older children were significantly more accurate in their reporting of the events of the fire evacuation. The younger preschoolers’ recollections were not guided by a script or causal mechanism, and as a result their stories and event narratives were more frequently devoid of a causal explanation and structure. Older preschoolers, however, paid greater attention to causality which therefore increased the temporal coherence of their narratives (Pillemer, 1992).

When older children and adults *over*-rely on their scripts, however, it can cause a developmental reversal. This can occur when an event does not accurately or completely fit into one’s script; in such a case, memory can be altered by the expectations that preceded. For example, consistent with over-reliance on what usually happens when a child visits the doctor, Ornstein et al. (1998) found 6-year-olds to be more likely to accurately report that they had their heart checked with a stethoscope at a medical appointment than 4-year-olds who did not possess as elaborate of a script for doctor visits. However, when an event fails to mimic a script, it can lead to false recollections, such as claiming that their heart had been checked with a stethoscope when this had not been part of the appointment. Hence, scripts (and more broadly the interconnectedness of knowledge) can lead to both normative and reverse trends depending on the characteristics of the to-be-remembered event and their conformity with expectations based on knowledge structures like scripts and schema.

### 2.3. Metamemory

Metamemory—or the introspective understanding of how memory functions and the ability to patrol the workings of one’s memory in order to monitor and regulate memory effectiveness—is another factor affiliated with age-related trends in memory accuracy. Researchers have charted age trends in the metamemorial processes involved in regulating memory and the role these play in recall and recognition memory accuracy (for early treatments, see Flavell et al., 1970; Kreutzer et al., 1975; Flavell and Wellman, 1977; Wellman, 1978). Studies by Flavell et al. (1970) have demonstrated normative age trends after evaluating young children’s abilities to estimate the number of words they can recall from a list, assess if and when they have committed a list to memory (i.e., “Tell us when you have memorized all of the words in the list”), recognize factors that could impede memory retrieval (e.g., studying for a test in a noisy room), and gauge the current status of their memory. Older children and adolescents possess a greater introspective awareness that enables them to better monitor their own memories and consequently intervene with appropriate strategies (see Schneider, 1999, for a review of age differences in metamemory knowledge).

As children begin to develop an overall mastery of metacognition and its affiliated processes, their overall memory performance improves (Schneider and Pressley, 1997). This is the essence of the normative developmental position. Researchers have documented a number of contextual factors that influence the efficacy of metamemorial processes. Namely, Ceci et al. (2010) found that the nature of the mental representation (how elaborately structured semantic knowledge is) influences the efficacy of metacognition. They found that when younger children’s representations are elaborate (e.g., of cartoon characters from shows they frequently watch), their metacognitive awareness is significantly enhanced, whereas when their representation is impoverished vis-à-vis older children—which is usually the case—their metacognition is less efficient. This is an illustration of an important principle: knowledge not only directly influences the recollective process, but it also moderates the efficiency of underlying processes that support memory such as strategies, metacognition, and monitoring.

### 2.4. Arousal

Every experience invokes some degree of “stress,” or *arousal*, best defined within this context as the degree of stimulation in terms of excitement, from low to high, produced while encoding stimuli (Gomes et al., 2013). Previously indistinguishable within literature, arousal is often examined in tandem with the *valence*, or amenity of a stimulus, which we address later in this review as a separate exogenous source of suggestion. The relationship between “emotion,” or “mood”—typically a combination of arousal *and* valence—and memory has been investigated in the past (e.g., Gardner, 1932; Redmount, 1959), but it is only within the past several decades that arousal and valence have been analyzed separately (Tellegen, 1985; Brainerd et al., 2008a,b), and even more recently that the relationship *between* the two has been examined (Brainerd et al., 2008a,b; Brainerd, 2018).

The literature has long held and continues to maintain that heightening arousal, valence, and/or “emotion” contributes to increased memorability for events relative to neutral events (Bradley et al., 1992), but simultaneously an increased risk to *inaccurate* memories (Gardner, 1932; Brainerd et al., 2008a,b). As specific effects of low vs. high arousal circumstances have been further investigated, studies have suggested that high arousal stimuli may encourage reliance on gists over verbatim memory (Brainerd et al., 2008a,b; Bookbinder and Brainerd, 2016). This suggests that similarly to the patterns of FTT, when gist-representations are over-relied upon, susceptibility to false memories will increase.

Developmentally, we know that children are less capable of emotional regulation (see Thompson, 1991 for a review), which may suggest normative developmental trends in memory accuracy. Compared to children, adults have significantly greater emotional regulation skills, and may therefore be less susceptible to allowing heightened arousal to produce false memories. However, if trends similar to those of FTT are truly reflected in the context of arousal, we may again observe developmental reversals as age increases one’s ability to create gist-representations and therefore *over-rely* on these associations.

### 2.5. Stereotypes

Depending on the situation, social factors such as stereotypes or security of attachment also have the potential to produce normative or reverse developmental memory trends. For example, Shapiro and Brooks (2018) found that recall of younger children (6-year-olds) showed higher rates of accuracy when thieves exhibited gender-role inconsistent characteristics than did older children for whom the stereotype about thieves being males was firmly established. However, when young children are preemptively exposed to stereotype information, they often demonstrate even higher rates of error than older children because of a combination of the stereotype and the lack of countervailing strategies. Leichtman and Ceci (1995) illustrated this normative trend by introducing preschoolers to a man named “Sam Stone” and presenting false stereotypic information about Sam’s clumsiness. The children were then asked suggestive questions such as “When Sam Stone got that [teddy] bear dirty, did he do it on purpose or was it an accident?,” and “Remember when Sam Stone ripped the book? Did he rip it on purpose, or on accident?,” (p. 577) when he had not made any messes or ripped any books. The youngest children (3- and 4-year-olds) produced many more stereotype-congruent false memories than did their older peers. This is because they lacked the ability to “source” their memories; their “memories” were not the result of retrieving actual observations of Sam Stone, but rather merely reporting what was congruent with a stereotype of being clumsy.

Thus, the effect of stereotypes on suggestibility is similar to the effect of knowledge or scripts; all can elevate memory when they are relevant, but they also can lower recall when they are misplaced. When the development of stereotype-knowledge itself follows a normative trend and young children have not yet acquired it, then younger children’s memories cannot be distorted when expectations and experience do not align; however, their memories also cannot be *served* when expectation and experience *would* accurately align, thereby neither exhibiting clear normative nor reverse trends. In contrast, once a child has successfully acquired stereotype-knowledge, as demonstrated in Ceci et al. (1995), young children may over-rely on their expectations derived from the stereotype and normative trends may emerge.

### 2.6. Attachment style

The relationship that a child has with its parent(s), with a particular emphasis on maternal attachment, has previously been identified as one of the few individual difference variables that consistently predicts suggestibility (see Bruck and Melnyk, 2004, for a review). The work of Chae et al. (2014, 2021) reported that younger children were no more susceptible to false memories/suggestibility than older children if two factors were present: (1) the context was distressing (employment of the “Strange Situation,” in which the parent leaves the child alone in an unfamiliar room for 5 min before being reunited), and (2) the child has a secure attachment to their mother. The idea behind this is that context (distressing vs. non-distressing settings) as well as individual differences in temperament and personality (attachment style) influence memory accuracy. This may be particularly relevant within the context of forensic interviews, where children may be alone with an unfamiliar interviewer under particularly stressful conditions for an extended period of time.

## 3. Endogenous sources of suggestion

### 3.1. Source misattribution

Another factor in play regarding memory accuracy is the ability to monitor where information came from. *Source monitoring* refers to the processes involved in making attributions about the origins of memories and beliefs (Johnson et al., 1993). A large literature documents that with some exceptions source monitoring ability increases from early childhood through adolescence and young adulthood, then declines at approximately age 50, thus resulting in an inverted U-shaped developmental function (Foley et al., 1983; Johnson et al., 1993; Fraser Parker, 1995). Especially in young children, this can take the form of misidentifying where they heard or learned information as well as difficulties distinguishing between real, experienced events vs. imagined events (e.g., Ackil and Zaragoza, 1995; Fraser Parker, 1995; Poole and Lindsay, 1995). This can have important legal consequences such as when a witness “remembers” having personally experienced something that someone else had told them about, or a witness confuses something enacted in therapy with having actually experienced it (Loftus, 1997).

As was the case with strategy development, myriad of contextual factors influences the efficacy of source monitoring, including several that moderate age differences, such as the familiarity of the interviewer (Quas et al., 2000). Specifically in a forensic context, interviews and conversations with police, attorneys, judges, juries, social workers, therapists, parents, friends, peers, teachers, and so on, can extend over many months (Ceci and Bruck, 1995). Therefore, it is important to understand children’s capacity to segregate conversations and maintain accurate source tracking so experts can be sure that children are aware of whether their statements are the result of direct observation vs. something told to them by a parent or former interviewer.

### 3.2. Inferential reasoning

Age differences in inferential reasoning can also play a role in memory accuracy. An extensive history dating into the 1970s bears on developmental differences in drawing inferences, gap-filling, and backward causal attributions (working backward from an event to its cause). As we have noted, associations between presented items and non-presented lures typically place older individual’s memory at a disadvantage (i.e., claiming they saw or heard semantically related lures), resulting in reversed age trends because younger children often lack knowledge of the associations, thus are not misled by them. For example, if participants are shown a photograph that induces them to make a false backward causal inference (e.g., after viewing a photo of a waiter mopping water at a table where a customer was seated, they later erroneously infer that they saw a photo of the customer spilling the glass of water), older children and adolescents will have more such false memories than younger children given their stronger causal knowledge structures and proficiency in back-filling (Lyons et al., 2010). However, in some cases inferential reasoning does not explicitly lure subjects into false information (e.g., generalizing that the water must have been spilled by *someone* for it to be there, despite not witnessing the incident).

### 3.3. Strategy acquisition

One of the most researched areas of memory development concerns strategy development, i.e., deliberately deployed procedures that are enacted to achieve a mnemonic end, such as rehearsal or creating reminders (Wellman, 1988). Nearly universally, these strategies are associated with age-normative trends. Older children and adults are more conscious of the value of implementing these strategies and are more capable of using them spontaneously, efficiently, and effectively in tandem with memory. However, despite the usefulness of these strategies, additional factors may counter or even reverse their beneficial effect. Here we analyze three of the most well-researched strategies for supporting memory—rehearsal, organization/clustering, and elaboration.

*Rehearsal* entails repeating (verbally or mentally) the item(s) to be remembered. The ability to implement rehearsal as a memory-support strategy develops over early childhood but appears to change with age. Young children have the ability to repeat words during a memory task (Flavell, 1966), but they do not appear to fully internalize the benefits of using this strategy to support memory until sometime between 2nd and 6th grade (Justice, 1985). Utilizing rehearsal strategies will often result in normative developmental trends although countervailing factors that are present in the same task are capable of attenuating or reversing the positive effect of strategies like rehearsal. For example, a task that would normally benefit from the use of rehearsal and therefore produce a normative trend because older individuals are more likely to have acquired it, may contain countervailing factors such as semantic associations, which work against older individuals, and could consequently cause reverse outcomes.

The *organization/classification* strategy refers to the grouping of items to be remembered into meaningful clusters or categories (e.g., fruits, animals, farm states). Recognition of the categorical structure of lists reduces the burden on memory by providing natural retrieval cues, i.e., the organizational structure itself. This strategy continues to demonstrate normative developmental trends, with organizational strategies not being implemented consistently until approximately age 8 (Best and Ornstein, 1986) despite the fact that even preschoolers demonstrate the capability to organize on the basis on semantic meaning such as grouping all fruits or animals (Corsale and Ornstein, 1980). But as one can infer from the aforementioned discussion on knowledge, this strategy can also impede memory and ultimately yield reverse developmental trends when the same semantic knowledge underpinning organizing items also leads to semantic confusions such as the false belief that a non-presented item had been presented.

*Elaboration* refers to the action of making visual or verbal connections between the items to be remembered or between these items and salient objects. Establishing interrelatedness between objects or ideas serves to establish meaningful connections during encoding, which can serve as memory cues during retrieval. Again, we find that this strategy can be deployed by young children when explicitly instructed to do so, however, like rehearsal, spontaneous use of it does not appear until adolescence (Pressley and Levin, 1977). Naturally, we may anticipate seeing normative developmental trends emerge as the ability to spontaneously implement elaborative techniques develops further. As one of many examples, Beuhring and Kee (1987) found age-related increases in performance on paired-associate tasks between 5th and 12th graders. Moreover, they concluded that 96 percent of this improvement could be accounted for by increased use of the elaboration strategy.

Conversely, one of the most prolific demonstrations of elaboration has been via the Deese, Roediger, and McDermott (DRM) task, which has formed the bedrock of evidence supporting developmental reversals. The DRM standardly entails presenting participants with lists of “target” words (e.g., mad, fear, hate, rage, temper) associated with a non-present “lure” word (anger) followed by recognition and recall tests. Manipulation of factors such as strength of the associated words, speed of presenting words, length of word list, time between list exposure and testing, implementation of memory strategies such as rehearsal, and so on, and their impact on rates of false memories have been previously explored (e.g., Hancock et al., 2003; Watson et al., 2004; Cann et al., 2011; Pardilla-Delgado and Payne, 2017).

A large body of literature investigating the DRM has long reflected the positive linear relationship between likelihood of mistakenly recalling the non-present lure word and age (e.g., Norman and Schacter, 1997; Tun et al., 1998; Balota et al., 1999; Holliday et al., 2011). These trends are likely reflective of the processes affiliated with FTT, and more specifically, age-related trends in spontaneous vs. suggestion-induced false memories. FTT and the DRM have both been associated with the production of *spontaneous* false memories, or memories produced without suggestion from the external environment (Otgaar et al., 2013).

This entire field of research is based in the idea of associative activation, or the triggering of related concepts in one’s mind based on exposure to an initial stimulus (Collins and Loftus, 1975; Otgaar et al., 2019). Again tying back to earlier discussion, with age typically comes the skillset of interconnecting ideas, increased knowledge, and greater ability to rely on elaboration skills; thus the strengthening of associative activation. It is when these methods coincide accurately and with experience and aid in memory storage and retrieval that normative developmental trends emerge. However, it is when expectations are broken and age coincides with an *over*-reliance on these associations that we observe developmental reversals.

## 4. Exogenous sources of suggestion

### 4.1. Valence

Another variable often studied in the context of the DRM is *valence*. As previously stated, valence refers to the amenity of a stimulus, or its *pleasantness*, ranging from positive to neutral to negative (Gomes et al., 2013). The DRM has been consistently used for examining effects of valence and/or arousal, largely given the numerous standardized lists established by experts [e.g., the Affective Norms for English Words (Bradley and Lang, 1999)]. As discussed earlier, increased arousal has been shown to heighten memorability of an event, but also potentially heighten reliance on gist traces over verbatim memories.

In contrast, valence has been shown to demonstrate a U-shaped relationship to memory, where higher valence, positive *or* negative, is shown to trigger greater memorability than neutral events (Bradley and Lang, 1999). As literature has moved further yet from seeing valence and arousal as one and begun to investigate the relationship between them, researchers have proposed that this U-shaped relationship may be attributable to the rise in arousal that results from increased valence, positive or negative (Brainerd and Bookbinder, 2019). We thereby continue to investigate age trends akin to those discussed in the context of arousal and anticipate that, situationally, normative or reverse developmental trends may emerge.

### 4.2. Suggestive techniques

Suggestive techniques include a large class of endogenous practices and exogenous procedures such as (mis)leading questions, imagery inductions, repetitions, false assertions, scripts, forced confabulations, forced-choice questions, and so on. Evidence of techniques such as these date back over a century, and their implications in suggestibility have been replicated across both individual studies and literature syntheses ever since (see Ceci and Bruck, 1993, for a review).

Of these techniques, misleading questioning is both one of the oldest on record and one of the most conceptually salient. For example, asking “Was the man’s hat black?” when the man was not wearing a hat is an explicitly misleading question. However, later research indicated that forensic interviewers need not make explicit suggestions in order to influence memory. Implicit suggestive strategies such as stereotype-guided recollection (Leichtman and Ceci, 1995), naturally occurring conversations between peers, or overhearing adult conversations (see Principe and Schindewolf, 2012, for a review) were also shown to produce robust misinformation effects, especially in preschoolers. The effects of this were shown to linger, with children continuing to reveal signs of distortion up to 1 year after exposure to false or misleading information (e.g., Peterson et al., 2001; London et al., 2009).

### 4.3. Suggestion-induced false memories

Our emphasis on evaluating children’s memory capabilities does not imply that adults are exempt from memory errors under suggestive questioning. Dating back to the 1990s with the work of Loftus and her colleagues, there is evidence that memories can not only be altered, but completely created and implanted (e.g., Loftus, 1993, 1997, 2003). This literature continued to expand, examining a number of ways to implant false memories, such as through word lists (e.g., the DRM) and cognitive tasks (e.g., the Brown-Peterson task), in which experts were able to implant false memories (e.g., Roediger and McDermott, 1995; Dodson and Schacter, 2001; Ghetti et al., 2002). The various suggestive techniques and factors previously discussed, such as suggestive questioning techniques have repeatedly been demonstrated to impact the prevalence of false memories across age groups (e.g., Ackil and Zaragoza, 1998; Garven et al., 1998). The overarching trends and likelihood of obtaining false memories do, however, remain based on those of the factors influencing them.

## 5. Legal relevance

The most direct implication of suggestibility and its impact on memory within the legal system can be seen in eyewitness testimony. Much of the research that has been done on memory and its legal implications has been motivated by cases in which children are called to present eyewitness testimony about criminal and custodial events (Ceci and Bruck, 1995; Poole and Lamb, 1998; Gordon et al., 2001; Ornstein and Haden, 2001).

Eyewitness testimonies are based on the autobiographical memory of a person who is alleged to have witnessed or participated in a crime. Already, it is crucial to be mindful of the myriad sources of suggestion aforementioned, particularly given the customarily negatively valenced and highly arousing nature of crime. In addition to the factors previously discussed that may impact memory prior to forensic interviewing, the interviews themselves may influence memory as well. Specifically, the strategies and techniques employed by law enforcement and social service officials to obtain information or a confession can be coercive or deliberately embellished with false information.

Existing or past interrogation strategies such as the Reid technique have been known to prey on one’s susceptibility to memory distortion and exacerbate the likelihood of eliciting a false confession (Kozinski, 2017). As demonstrated in research by Loftus and Pickrell (1995) and Loftus (1996, 1997), false memories are relatively simple to implant, and further studies such as those by Otgaar and his associates (e.g., Otgaar et al., 2021) have shown that increased levels of suggestibility yield higher rates of false confessions. This underscores the necessity to understand how chronological age corresponds with trends in memory development.

Given the influence of the factors discussed throughout this paper, there is no clear indication that the legal testimony of young children need be discarded altogether. It may prove advantageous to take extra precautions when interviewing young, especially preschool-aged, children, considering that age-related differences *do* exist regarding susceptibility to suggestion. But the data indicate that children still are capable of retrieving memories with a great deal of accuracy (Ornstein et al., 1992). Moreover, as we have argued throughout this paper, the possibility of reverse developmental trends underscores the inadvisability of automatically assuming that children’s testimony is inferior to that of adults in all circumstances.

## 6. Conclusion

Experts have historically assumed the normative developmental position, believing that as age increases, susceptibility to memory distortions decreases. The underdevelopment of children’s neurobiological architecture involved in source monitoring, tracking, and response inhibition; the underdevelopment of certain cognitive functions (e.g., strategies, associative knowledge, and inferential reasoning); and myriad social influences on young children (e.g., desire to please authority figures, yes-bias, insecure attachment, and peer conformity) undermines their report accuracy. However, this is not to say that there are not cases in which children—even young children—demonstrate superior memory performance to adults. As we have shown, there are times when older individuals’ superior semantic knowledge and inferential skills will taint their recollections in ways that do not jeopardize younger children whose lack of knowledge insulates them.

Thus, our literature review reveals that memory trends do not ineluctably follow normative patterns, but rather that there are factors and circumstances that *have the potential* to yield reverse trends; notably, when older individuals possess greater knowledge and comprehension of the world around them that can, in certain contexts, reduce memory accuracy. Older individuals’ greater ability to optimize memory-support strategies and greater knowledge can, depending on the specifics of the situation, result in greater memory accuracy, but as demonstrated, this is not always the case—particularly when the greater knowledge leads to false associations. Thus, some of the very acquisitions that lead to accurate recollection can lead to inaccurate recollection when they promote spreading activation of non-observed events.

Overall, we see evidence that when exogenous sources of suggestibility are controlled for and memory aids are employed correctly, age trends are neither clear nor consistent with regards to susceptibility to suggestion or accuracy. The primary dilemma historically has been the lack of distinction between children’s true memory accuracy and their reporting accuracy, largely resulting from a lack of control for suggestive factors. As we have gained insight into an extensive list of endogenous and exogenous sources of suggestibility and now further expounded on the conditions under which these factors are subject to influencing developmental trends, the primary responsibility of continuing research will be to investigate how these factors impact one another.

Given the nature of human memory and its variability, susceptibility to memory distortion is context-specific. A task for future researchers will be to formalize the boundary conditions, which will require a dedicated program of research. The ways in which the factors that we have identified interact across every juncture of the memory process illuminates *contingent* age-related trends. The numerosity of these factors, their diversity within the existing literature, and the complexities of their interactions pose a challenge to creating a formal quantitative model that leads to specific expectations. As additional research regarding the sources of memory influence unfolds, it may become easier to create more dynamic and explicit models for predicting age-related trends in memory.

The goal of this review was to synthesize existing studies in such a way that developmental contrasts can be seen across the literature as a whole. In doing so, we have proposed a preliminary outline that can explain and predict when normative vs. reverse developmental outcomes will be observed. This review supports the notion that children, including young children, should not be automatically discredited within the legal setting purely on the basis of their age. When children are the victims of crime or are the only eyewitness, their testimony can be extremely valuable, and no evidence exists that they are invariably inefficacious. However, to harness children’s potential, it is important that we utilize this information and ensure that participants in the legal system (social workers, law enforcement officers, therapists, attorneys) are not contributing to endogenous or exogenous sources of suggestibility.

## Author contributions

SC conceived and conceptualized the idea. BR wrote the manuscript with support from I-AS and SC. BR, I-AS, and SC contributed intellectually to the content within this article, as well as reviews and revisions. I-AS organized external affairs (e.g., funding). All authors contributed to the article and approved the submitted version.

## Funding

Publishing fees were endowed in-part by the Cornell Open Access Publishing (COAP) Fund.

## Conflict of interest

The authors declare that the research was conducted in the absence of any commercial or financial relationships that could be construed as a potential conflict of interest.

## Publisher’s note

All claims expressed in this article are solely those of the authors and do not necessarily represent those of their affiliated organizations, or those of the publisher, the editors and the reviewers. Any product that may be evaluated in this article, or claim that may be made by its manufacturer, is not guaranteed or endorsed by the publisher.
